# Supplementary material for: Effect of Alpha-Lipoic Acid Supplementation on Low-Grade Squamous Intraepithelial Lesions—Double-Blind, Randomized, Placebo-Controlled Trial
Source: Healthcare (Basel). 2022 Dec 2;10(12):2434. doi: 10.3390/healthcare10122434 (PMC9778332; doi:10.3390/healthcare10122434)
Supplement: Supplementary file 1 [file healthcare-10-02434-s001.zip › Table S2.pdf]

	PATIENT CODE	age	BMI (kg/m2)	smoking	HPV 1	SIL 1	hsCRP 1	FI 1	SE 1	HPV 2	SIL 2	hsCRP 2	FI 2	SE 2	FRUIT	VEGETABLE	ENERGY	MEAT	ANIMAL PROTEIN
2	2	51	25.18	NE	Ne	2	0,31	2,3	7	Ne	1	0,42	2,4	9	3.07234	3.6128	14450.1	3.02348	39.0294
3	3	33	20.44	NE	Ne	2	0,99	2,2	20	Ne	2	1,86	2,5	23	5.64372	1.89709	1860.73	3.51165	23.7277
4	4	30	18.44	NE	Da	2	0,0	2,2	4	Da	1	0,18	2,1	8	4.3022	2.79232	1985.5	1.63772	24.0511
5	5	52	32.6	NE	Da	2	2,38	3,6	18	Da	1	1,55	2,8	14	3.68475	23.9309	6956.49	13.8072	85.9607
6	6	32	28.9	NE	Ne	2	2,01	3,0	15	Ne	1	1,54	2,6	13	0.52999	3.6507	1869.47	3.32583	17.1608
7	7	34	19.84	NE	Ne	2	0,49	2,4	6	Ne	1	0,48	2,3	14	4.86389	4.31501	2207.29	3.33843	25.0675
8	8	40	26.73	NE	Ne	2	0,47	3,5	16	Ne	1	1,59	5,3	31	1.46386	12.9377	5268.24	5.79501	65.1625
9	9	43	26.96	NE	Da	2	0,96	2,7	7	Da	1	0,21	2,4	4	1.34839	5.48718	4541.81	8.65472	74.2221
10	10	42	25.61	DA	Da	2	0,26	3,1	12	Da	2	0,39	3,8	8	3.22326	6.71769	2128.28	2.54476	18.9041
11	11	43	27.94	NE	Ne	2	3,49	3,1	27	Ne	1	4,30	3,7	16	6.52463	5.61961	2856.01	4.53522	36.0483
12	12	42	28.65	NE	Ne	2	0,97	4,4	26	Ne	1	1,36	5,4	34	2.44368	5.80736	4468.16	11.0546	67.005
13	13	49	32.42	NE	Ne	2	6,67	5,0	25	Ne	1	4,55	5,4	23	1.64206	4.01605	3079.62	6.89732	33.6733
14	14	23	23.72	DA	D	2	2,07	3,5	14	Da	1	0,47	2,4	12	0.57251	1.80716	7056.96	13.2529	44.8226
15	15	43	27.33	DA	Da	2	0,97	3,4	15	Da	1	0,52	3,3	12	0.15205	3.44192	3646.05	12.9128	49.4156
16	16	42	23.81	NE	Ne	2	1,23	4,2	21	Ne	1	0,84	3,1	16	3.15962	5.91884	4455.51	4.28642	56.0624
17	19	45	32.03	NE	Da	2	5,39	4,9	36	Da	1	2,50	4,7	28	3.72463	6.23739	4630.54	6.33672	62.3453
18	20	33	26.33	NE	Ne	2	2,33	4,2	30	Ne	1	2,20	3,4	27	0.48827	4.17438	3952.78	4.19508	20.4024
19	21	47	29.38	NE	Da	2	11,29	3,9	26	Da	2	2,33	3,2	25	4.10082	4.60601	3874.55	13.1018	52.7017
20	22	47	26.83	NE	Da	2	1,79	4,6	27	Da	1	1,75	4,5	22	10.1273	4.62745	4097.36	4.03131	46.8841
21	23	36	25.16	DA	Da	2	1,08	3,7	15	Da	1	1,13	3,7	14	3.54099	4.33442	3477.34	5.01394	59.6603
22	24	44	23.53	NE	Ne	2	2,04	4,0	23	Ne	1	1,44	3,9	15	2.7376	3.35764	3778.94	4.14469	22.5214
23	25	50	26.29	NE	Ne	2	0,22	4,4	20	Ne	1	0,20	3,9	18	3.53846	2.86762	2582.53	2.3306	27.8785
24	28	41	17.11	NE	Ne	2	2,92	3,1	25	Ne	1	0,38	2,2	18	1.54278	0.47392	1288.78	8.69251	40.187
25	29	22	28.04	NE	Da	2	2,87	4,7	26	Da	1	2,45	3,7	22	4.22619	0.26229	8143.9	34.1402	14.4004
26	30	43	26.22	NE	Ne	2	1,81	4,4	8	Ne	1	1,65	3,8	4	2.44976	6.34138	5604.25	3.22505	35.66
27	31	51	25.56	NE	Ne	2	1,25	4,3	15	Ne	1	0,82	3,4	11	3.65385	3.36827	1941.51	3.03608	41.6886
28	32	39	24.98	DA	Ne	2	2,62	3,9	25	Ne	1	1,32	3,7	22	3.93197	6.34288	3292.44	4.33366	39.3639
29	33	46	33.06	NE	Da	2	1,94	3,0	19	Da	1	1,12	2,6	16	1.58027	0.5766	895.423	2.14163	13.4787
30	34	24	20.81	NE	Ne	2	1,73	5,7	30	Ne	1	0,48	3,8	25	10.1446	3.79482	8198.11	5.16512	37.9317
31	35	39	21.72	DA	Ne	2	1,06	5,3	24	Ne	2	2,80	5,3	25	8.29286	6.53183	4365.37	27.0602	161.708
32	36	21	31.35	DA	Ne	2	11,54	5,2	28	Ne	2	20,23	5,4	30	0.79945	3.08852	1959.51	4.74939	33.7
33	37	31	23.14	NE	Da	2	0,86	3,4	20	Da	1	0,22	2,7	16	3.07658	4.57436	4476.56	5.46746	52.5603
34	39	38	23.94	DA	Da	2	0,98	2,7	27	Da	1	0,46	2,4	20	0.50493	0.5316	2516.79	0	20.1611
35	40	50	23.26	NE	Ne	2	3,07	3,6	30	Ne	1	2,66	3,5	25	0.06207	2.14366	586.771	2.51957	25.852
36	41	25	18.94	NE	Ne	2	1,02	3,8	22	Ne	2	1,70	4,0	25	2.92268	3.59431	2942.14	2.23612	23.1423
37	42	49	28.69	NE	Ne	2	0,59	2,5	15	Ne	2	0,67	2,6	20	5.41387	10.7232	3157.55	0.5795	40.3391
38	43	37	27.97	NE	Ne	2	2,39	3,2	14	Ne	2	2,47	3,6	20	6.24904	3.86821	2538.23	12.3963	83.7028
39	44	43	25.95	NE	Ne	2	3,85	3,7	17	Ne	2	3,94	4,6	26	1.89574	1.65776	2167.98	0.68028	16.4911
40	45	45	22.41	NE	Ne	2	2,36	3,2	12	Ne	2	2,55	3,7	20	4.42179	2.68141	3286.53	3.17466	55.6054
41	46	28	20.34	NE	Ne	2	0,33	2,4	5	Ne	2	0,35	2,4	10	2.39312	5.46505	3831.22	9.90191	91.5198
42	47	31	22.68	NE	Ne	2	0,74	2,4	12	Ne	2	0,82	2,6	18	6.67016	3.58134	3233.16	4.1069	35.2784
43	48	31	21.14	NE	Ne	2	0,14	2,4	11	Ne	2	0,58	3,1	15	1.26833	2.05173	5873.72	1.82669	146.538
44	49	44	27.34	DA	Da	2	0,68	3,6	14	Da	2	1,08	3,8	22	5.897	3.5422	4215.85	1.53694	22.0414
45	50	27	28.65	DA	Ne	2	1,26	3,4	22	Ne	2	1,29	3,5	25	0.85077	2.3303	3050.74	7.81696	41.7945
46	51	46	28.2	NE	Ne	2	5,11	3,3	28	Ne	2	6,04	3,8	30	8.04533	2.02369	3385.06	2.18888	47.8101
47	52	35	21.05	NE	Da	2	0,47	2,3	15	Da	2	1,67	4,0	24	3.46154	4.20865	3811.06	1.7637	26.1419
48	54	34	21.97	NE	Da	2	0,15	2,2	17	Da	1	0,15	2,3	15	6.20945	8.76982	4041.28	11.8924	77.1413

	A	B	C	D	E	F	G	H	I	J	K	L	M	N	O	P	Q	R	S
49	56	50	27.18	NE	Ne	2	1,80	3,6	25	Ne	2	2,09	4,3	27	0.1163	1.96014	1060.41	5.29109	29.9186
50	57	45	27.28	NE	Ne	2	0,11	2,4	10	Ne	2	0,13	2,9	12	2.15239	3.26874	4842.01	2.78412	44.77
51	58	37	21.88	NE	Da	2	0,88	4,4	22	Da	2	0,92	4,6	20	0.46981	11.0555	5775.86	14.7899	74.3093
52	59	47	24.98	NE	Da	2	1,06	4,3	23	Da	2	1,11	4,5	26	2.24495	2.24759	1155.78	3.27544	45.5686
53	60	46	26.13	DA	Da	2	0,64	3,4	23	Da	2	0,65	4,0	27	0.63363	2.59107	1229.23	4.53522	34.8484
54	62	42	28.41	DA	Da	2	1,06	3,4	28	Da	2	1,16	3,5	27	5.84136	4.76298	4260.58	7.91145	80.2704
55	63	27	31.89	DA	Ne	2	12,44	5,3	30	Ne	2	13,78	5,4	29	5.81406	9.13721	4662.44	9.14603	70.7776
56	65	45	20.76	NE	Ne	2	1,78	4,9	20	Ne	1	1,06	4,5	16	1.32927	4.23424	1112.65	0.55431	11.5624
57	67	32	23.11	NE	Ne	2	0,48	3,3	15	Ne	2	0,57	3,9	17	3.64211	2.54677	3819.3	1.55583	22.8052
58	68	50	23.88	NE	Ne	2	0,35	2,9	16	Ne	2	0,36	3,3	18	2.62798	4.22371	1929.83	2.3558	32.5398
59	69	52	26.17	DA	Da	2	1,01	4,4	20	Da	2	1,05	4,7	23	0.09566	0.34774	8486.66	0.39053	20.3478
60	70	30	22.49	NE	Da	2	12,39	5,3	30	Da	2	13,03	5,9	28	2.48943	2.43512	797.552	4.27067	13.5068
61	71	48	25.53	NE	Ne	2	0,06	2,3	10	Ne	2	0,09	2,5 g#L	12	2.13861	3.82371	1389.72	7.6091	63.9786
62	72	47	20.83	NE	Da	2	1,76	4,2	20	Da	1	0,89	3,4	10	10.2015	3.39643	3160.57	1.41096	27.8934
63	73	46	21.09	DA	Ne	2	0,03	2,2	12	Ne	2	0,15	2,5	15	10.9643	5.31697	2192.7	0.97633	10.2835
64	74	35	16.98	DA	Ne	2	0,85	2,8	20	Ne	2	0,89	3,0	25	3.12075	4.40507	1886.8	2.51957	19.383
65	75	50	27.33	NE	Da	2	1,37	4,3	31	Da	1	1,5	5,0	34	2.39286	7.00096	5108.45	4.63601	45.0232
66	76	48	25.1	NE	Da	2	7,92	4,3	35	Da	2	8,0	5,0	36	3.58499	3.44123	1471.86	2.51957	27.6804
67	77	30	18	NE	Da	2	0,17	2,6	8	Da	1	0,12	2,4	7	7.95879	5.08781	3530.42	2.70854	56.7785
68	78	53	26.53	NE	Ne	2	1,77	4,3	16	Ne	1	1,51	4,1	10	3.92924	2.03029	4353.07	4.32106	40.1695
69	79	28	21.83	NE	Ne	2	0,71	2,1	10	Ne	2	0,74	2,5	14	3.95489	17.6197	17331.7	7.44533	84.1714
70	80	31	21.8	NE	Ne	2	0,36	3,4	12	Ne	2	0,40	4,0	16	5.47411	0.47278	1634.28	0.99523	16.4508
71	81	27	19.13	NE	Ne	2	0,14	2,2	10	Ne	2	0,25	2,5	13	7.85774	1.64181	2762.01	7.15558	45.3079
72	82	23	23.18	NE	Da	2	1,08	2,5	18	Da	2	1,11	2,8	20	5.46008	6.41912	4268.18	8.55394	56.0946
73	83	28	20.55	NE	Ne	2	0,16	2,6	12	Ne	2	0,20	2,8	13	2.64516	1.91549	1321.86	3.70377	26.4317
74	84	27	23.89	DA	Da	2	2,12	2,2	15	Da	2	2,18	2,7	17	2.30178	1.97942	2089.07	2.30541	21.3469
75	85	28	21.14	NE	Ne	2	0,11	2,2	8	Ne	2	0,70	2,9	14	1.0591	2.19707	1645.99	3.86124	16.6394
76	86	28	26.99	NE	Da	2	0,59	3,3	16	Da	2	0,64	3,8	17	2.46232	2.22687	3459.5	12.0561	95.6813
77	87	29	18.94	DA	Da	2	0,72	1,9	13	Da	2	1,2	2,5	17	1.89917	3.7634	2326.8	4.62341	29.6184
78	88	24	23.6	DA	Ne	2	0,70	2,1	7	Ne	2	1,2	2,5	9	0.33142	0.85414	2192.14	1.53694	40.8873
79	89	41	20.94	DA	Ne	2	0,13	2,7	15	Ne	1	0,21	2,5	10	5.08314	2.96282	4321.16	1.25978	26.7426
80	90	41	26.78	NE	Da	2	5,86	5,1	25	Da	2	6,2	5,5	28	2.24596	9.00526	3956.84	2.68649	29.5772
81	91	38	24.17	NE	Da	2	0,86	3,9	20	Da	2	1,1	4,2	24	4.5642	3.29588	3611.86	4.03131	47.9567
82	92	51	22.72	NE	Ne	2	0,48	2,0	14	Ne	2	0,54	2,5	19	1.75627	2.57796	2180.31	7.48312	67.8457
83	93	50	21.48	NE	Da	2	1,15	4,0	23	Da	2	1,5	4,5	25	3.56539	4.33494	2758.44	6.70835	58.8988
84	94	31	18.59	NE	Ne	2	0,46	2,2	10	Ne	1	0,3	1,8	8	10.336	11.3218	3536.42	7.23116	55.4483
85	95	51	18.59	NE	Da	2	2,79	4,4	28	Da	1	1,9	3,8	23	9.56894	8.23458	3678.55	6.23593	59.1222
86	96	29	21.85	NE	Da	2	1,52	3,3	20	Da	1	0,90	2,6	15	5.54287	0.60002	956.635	0.55431	9.88335
87	97	49	24.03	NE	Da	2	5,68	4,7	29	Da	1	3,9	3,1	24	3.65935	6.25343	2044.28	2.36839	18.3537
88	98	51	25.59	DA	Ne	2	0,63	4,9	23	Ne	1	0,30	3,5	19	6.80735	4.35282	1836.44	3.42661	27.7967
89	99	46	24.62	NE	Ne	2	0,68	4,1	26	Ne	1	0,41	3,2	23	7.24542	3.14198	2896.19	7.25636	121.684
90	100	46	25.65	DA	Ne	2	1,96	4,2	22	Ne	1	0,7	3,1	17	0.68989	2.68479	1305.52	3.11797	27.6598
91																			
