# Supplementary figures and images for: Effect of Alpha-Lipoic Acid Supplementation on Low-Grade Squamous Intraepithelial Lesions—Double-Blind, Randomized, Placebo-Controlled Trial
Source: Healthcare (Basel). 2022 Dec 2;10(12):2434. doi: 10.3390/healthcare10122434 (PMC9778332; doi:10.3390/healthcare10122434)

## CONSORT 2010 Flow Diagram

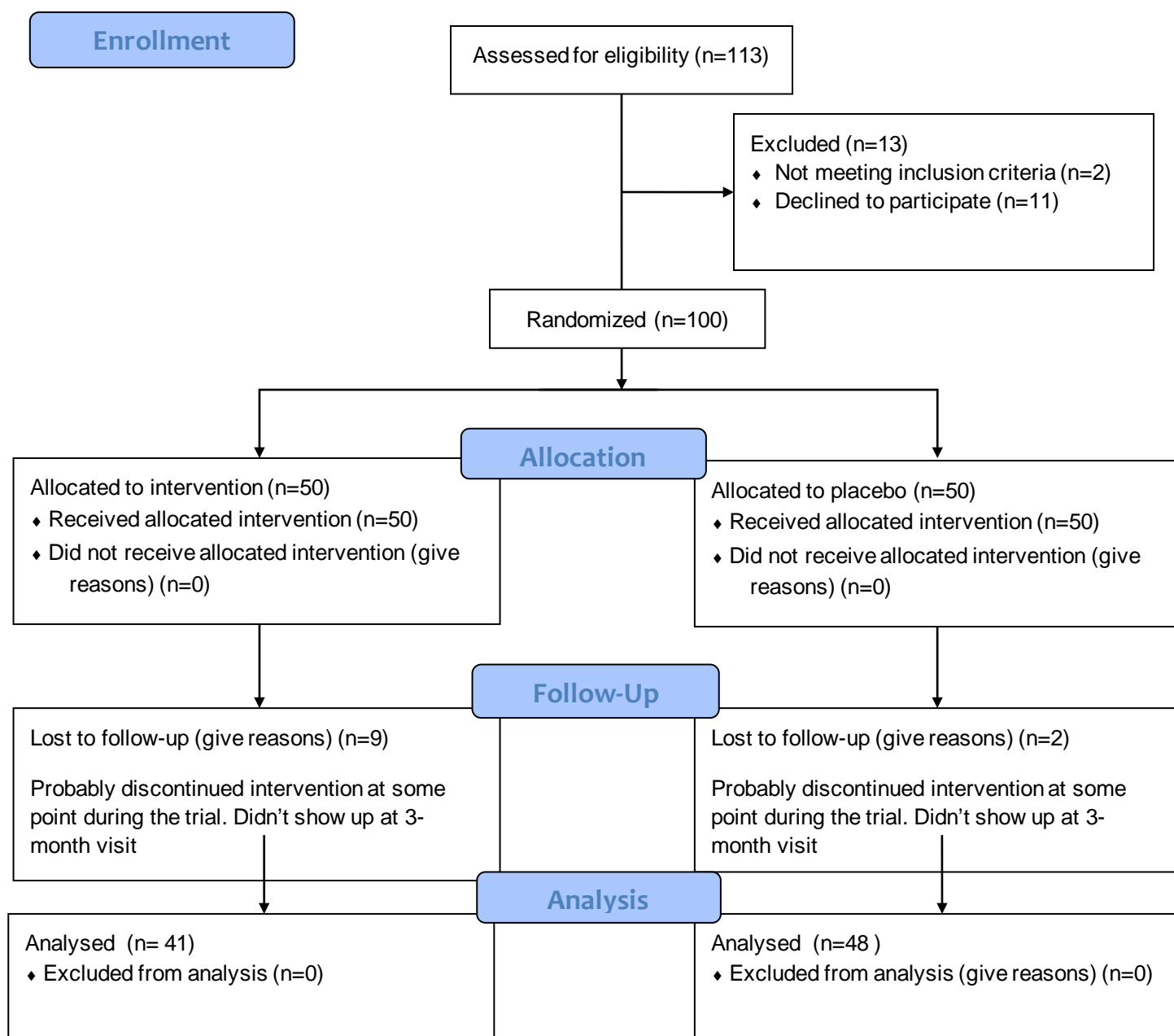

Supplement: Supplementary file 1 [file healthcare-10-02434-s001.zip › Figure S1.pdf]
